# Association between oxidative balance score and methylation cycle biomarkers in US adults: insights from the national health and nutrition examination survey

**DOI:** 10.3389/fnut.2025.1526025

**Published:** 2025-04-28

**Authors:** Xuna Liu, Yiwen Wang

**Affiliations:** ^1^Shaanxi Provincial People's Hospital, Xi’an, China; ^2^Xi'an International Medical Center Hospital Affiliated to Northwest University, Xi’an, China

**Keywords:** methylation cycle, oxidative balance score, folate, vitamin B, homocysteine, methylmalonic acid, NHANES

## Abstract

**Background:**

Oxidative stress(OS) is implicated in various diseases, and the methylation cycle plays a critical role in numerous biological processes including DNA methylation and antioxidant defense. We aimed to investigate the relationship between oxidative balance score (OBS) and methylation cycle.

**Materials and methods:**

The study enrolled 28,061 adults ≥20 years old from the NHANES 2003–2014. Methylation cycle biomarkers included serum folate, RBC folate, vitamin B6, vitamin B12, homocysteine (HCY), and methylmalonic acid (MMA). OBS was scored by 20 dietary and lifestyle factors. We employed weighted linear regression and restricted cubic spline (RCS) models to analyze the correlations among OBS, and methylation cycle.

**Results:**

In a nationally representative cohort of US adults (mean age: 47.04 ± 0.25 years; 51.76% female), OBS demonstrated significant associations with key methylation cycle biomarkers. After adjusting for potential confounders, OBS exhibited a positive association with serum folate, RBC folate, vitamin B6, and vitamin B12, a negative correlation with HCY (all P for trend < 0.001), and no correlation with MMA (P for trend = 0.07). Higher dietary OBS was correlated with increased levels of serum folate, RBC folate, vitamin B6, and vitamin B12, and decreased levels of HCY (all P for trend < 0.001). Similarly, a higher lifestyle OBS corresponded to increased serum folate, vitamin B6, and vitamin B12, as well as decreased HCY (all P for trend < 0.05). Stratified analyses demonstrated that OBS had a strong positive correlation with serum folate, RBC folate, vitamin B6, and vitamin B12, and a strong negative correlation with HCY. Additionally, the negative correlation between OBS and MMA was observed in the elderly population (≥ 60 years old) when stratified by age. RCS regression analysis revealed that with the increase in OBS, serum folate, RBC folate, vitamin B6, and vitamin B12 also increased, while HCY and MMA decreased.

**Conclusion:**

Our findings demonstrate a significant relationship between OBS and the methylation cycle. Higher OBS is positively associated with folate and vitamins B6/B12, and negatively with HCY.

## 1 Introduction

Oxidative stress (OS), arising from an imbalance between reactive oxygen species (ROS) production and the body’s detoxification capacity, is implicated in various diseases, including cardiovascular diseases (CVD), cancer, diabetes mellitus (DM), and neurodegenerative disorders (1, 2). The oxidative balance score (OBS) quantifies the cumulative effects of pro-oxidant and antioxidant factors within an individual’s biological profile, reflecting both dietary intake and lifestyle elements for a holistic view of the redox state (3).

Central to many biological processes is one-carbon metabolism, a biochemical pathway critical for DNA synthesis, repair, and epigenetic regulation (4). This pathway relies on the interplay of several key metabolites, including serum folate, red blood cell folate, vitamin B6, vitamin B12, homocysteine (HCY), and methylmalonic acid (MMA) (5). Folate plays a central role in the remethylation of HCY to methionine, while vitamins B6 and B12 act as essential co-factors in these metabolic processes (6). One-carbon metabolism is tightly coupled with the methylation cycle, ensuring a continuous supply of methyl groups for DNA and RNA synthesis, as well as epigenetic modifications that regulate gene expression (7). Epidemiological evidence has linked disruptions in one-carbon metabolism and the methylation cycle to various pathologies. Elevated HCY levels, for instance, have been consistently associated with increased cardiovascular risk (8), while abnormal MMA concentrations may indicate vitamin B12 deficiency or impaired methylation capacity (9). These findings highlight the critical role of one-carbon metabolism in maintaining health and preventing disease.

Despite the established importance of both oxidative balance and the methylation cycle in human health, few studies have systematically explored their interrelationship. Most previous research has focused on isolated components rather than examining the comprehensive association between OBS and the methylation cycle as a whole. This gap in knowledge limits our understanding of how lifestyle and dietary factors collectively influence this critical metabolic pathway. This study aimed to investigate the relationship between OBS—a composite measure of pro- and antioxidant exposures—and biomarkers of the methylation cycle (folate, B vitamins, homocysteine, and MMA) in a nationally representative US adult population. We hypothesized that higher OBS, reflecting greater antioxidant capacity, would correlate with optimal methylation cycle function.

## 2 Materials and methods

### 2.1 Study population

Participants in this study were from NHANES 2003–2014. NHANES was approved by the National Center for Health Statistics ethics review committee, and all participants submitted written informed consent. All procedures for this study were conducted following relevant guidelines and regulations.^1^ Of the 61,087 subjects in the 6 NHANES cycles, subjects were excluded if they were (1) under 20 years of age (*n* = 27,585), (2) missing OBS data (*n* = 3,504), (3) missing education data (*n* = 31), and (4) missing methyl cycle markers (*n* = 1,906). Ultimately, a total of 28,061 subjects were included in this study.

### 2.2 Measurement of oxidative balance score

OBS was determined by evaluating 16 nutrients and 4 lifestyle elements, comprising 5 pro-oxidants and 15 antioxidants, based on established knowledge linking OS with nutrients and lifestyle factors (10). The dietary intake of these 16 nutrients—such as dietary fiber, carotene, riboflavin, niacin, vitamin B6, total folate, vitamin B12, vitamin C, vitamin E, calcium, magnesium, zinc, copper, selenium, total fat, and iron—was ascertained from the first dietary review session. The lifestyle factors included physical activity, body mass index (BMI), alcohol consumption, and smoking, with smoking intensity quantified by cotinine levels. Among these factors, total fat, iron, BMI, alcohol consumption, and smoking were classified as pro-oxidants, while the remainder were viewed as antioxidants. Adopting the calculation approach for OBS as outlined by Zhang et al., alcohol consumption was stratified into three categories: heavy drinkers (≥15 g/day for women and ≥30 g/day for men), non-heavy drinkers (0–15 g/day for women and 0–30 g/day for men), and abstainers, who were allocated scores of 0, 1, and 2, respectively (10). Subsequently, the remaining components were segregated by gender and divided into tertiles, with antioxidants receiving scores ranging from 0 to 2 for groups 1 to 3 and pro-oxidants receiving scores from 2 to 0 for groups 1 to 3 (details are shown in Supplementary Table S1) (10). An elevated OBS indicates greater antioxidant exposure. Previous studies have explored the relationship between OBS and a variety of diseases, including stroke, diabetes, and sarcopenia (11–13).

### 2.3 Measurement of methylation cycle biomarkers

Informed by prior studies, we chose serum folate, RBC folate, vitamin B12, HCY, and MMA as indicators of methyl cycle activity (14). Recognizing the significance of vitamin B6 in this cycle, we incorporated it as an additional marker. Due to variations in the available measurements across different years in the NHANES database, serum folate and RBC folate data were extracted from NHANES cycles 2007–2008, 2009–2010, 2011–2012, and 2013–2014; vitamin B6 data were sourced from 2007 to 2008 and 2009 to 2010; vitamin B12 and MMA data were taken from 2011 to 2012 and 2013 to 2014; and HCY data were selected from 2003 to 2004 and 2005 to 2006. Analysis for each marker was performed in its respective year of availability. All samples underwent standardized processing, and preservation, and were shipped to the Division of Environmental Health Laboratory Sciences at the National Center for Environmental Health, CDC for analysis. Folates and vitamin B12 levels were measured using the Quantaphase II Folate/Vitamin B12 radioassay kit from Bio-Rad Laboratories, Hercules, CA. Vitamin B6 levels were determined through High-Performance Liquid Chromatography (HPLC). HCY levels were quantified using an automated fluorescence polarization immunoassay from Abbott Diagnostics, Abbott Park, IL. MMA concentrations were analyzed with gas chromatography-tandem mass spectrometry (GC–MS/MS), with equipment from Hewlett-Packard, San Fernando, CA. These state-of-the-art analytical techniques ensure precise and dependable measurement of the methyl cycle markers assessed.

### 2.4 Covariates ascertainment

Within our investigation, the variables controlled for included age, sex (female and male), race (Mexican American, non-Hispanic black, non-Hispanic white, other Hispanic, and other races), education level (<high school, high school, and >high school), marital status (married/living with a partner, never married, separated/divorced/widowed), and poverty-to-income ratio (PIR, categorized as<1.3, 1.3–3.5, >3.5, No recode). Additionally, we accounted for the presence of hypertension (no, yes), DM (no, yes), CVD (no, yes), hyperlipidemia (no, yes), and cancer (no, yes). Hypertension, DM, and hyperlipidemia were identified through a combination of direct measurements, medication use, and self-reported information, while CVD and cancer were ascertained by self-report. The quality of the overall diet was evaluated using the 2015 Healthy Eating Index (HEI) and the total energy intake (15).

### 2.5 Statistical analysis

Individual sample weights were determined according to the NHANES-recommended dietary day one sample weight (WTDRD1), taking into account the survey’s multistage probability sampling design. In the analysis of baseline characteristics, continuous variables were expressed as weighted means with standard errors, while categorical variables were shown as sample sizes with weighted percentages. To compare variable characteristics across different OBS groups (quartiles), we conducted univariate analyses using ANOVA for weighted means of continuous variables and the Rao-Scott *χ*^2^ test for weighted percentages of categorical variables, aiming to profile the overall study population. OBS quartiles (Q1–Q4) were defined based on the distribution of scores, with Q1 representing the lowest 25% and Q4 the highest 25% of OBS values.

For the investigation of associations between OBS quartiles and methylation cycle biomarkers—serum folate, RBC folate, vitamin B6, vitamin B12, HCY, and MMA—we utilized weighted linear regression models. Three models were employed: the crude model without any adjustments, Model 1 adjusted for age, race, education, marital status, and PIR, and Model 2 further adjusted for HEI, total energy intake, hypertension, DM, CVD, hyperlipidemia, and cancer. We also analyzed dietary and lifestyle OBS components separately using weighted linear regression. Stratified analyses explored subpopulation differences by various factors, including age group, sex, race, education, marital status, PIR, hypertension, CVD, diabetes, hyperlipidemia, and cancer, with multiplicative interaction tests evaluating interactions between stratification factors and OBS. Additionally, restricted cubic spline (RCS) analysis, adjusting for age, sex, race, education, marital status, PIR, HEI, total energy intake, hypertension, DM, CVD, hyperlipidemia, and cancer, confirmed the associations between OBS components and methylation cycle biomarkers.

All statistical analyses were conducted using R version 4.4.1 (R Foundation for Statistical Computing, Vienna, Austria), and statistical significance was set at a two-sided *p* < 0.05.

## 3 Results

### 3.1 Baseline characteristics

The demographic characteristics of participants categorized by OBS quartiles are presented in Table 1: the average age of the individuals was 47.04 years with a standard error of 0.25 years, and 51.76% of them were female. The majority of the subjects identified as non-Hispanic white, constituting 69.99% of the sample. Compared to the lowest OBS quartile, participants in the highest OBS quartile had more non-Hispanic White, more individuals with married/living with a partner, higher education, higher wealth, higher HEI, higher total energy intake, higher serum folate, higher RBC folate, higher vitamin B6, lower HCY, lower MMA, and fewer co-morbidities, including hypertension, DM, CVD, and hyperlipidemia.

**Table 1:** Baseline characteristics of the study population by quartiles of the OBS.

Variable	Total	Q1	Q2	Q3	Q4	*P*-value
Age, y, mean (SE)	47.04 (0.25)	48.05 (0.31)	47.64 (0.37)	46.94 (0.35)	45.75 (0.40)	< 0.0001
Age, *n* (%)						< 0.0001
20–39	9,738 (37.06)	2,142 (35.10)	2,363 (36.41)	2,572 (36.47)	2,661 (39.89)	
40–59	9,019 (38.06)	2,138 (36.55)	2,254 (36.81)	2,423 (39.30)	2,204 (39.21)	
≥60	9,304 (24.88)	2,813 (28.36)	2,510 (26.77)	2,247 (24.23)	1734 (20.90)	
Sex, *n* (%)						0.38
Female	14,391 (51.76)	3,521 (52.67)	3,651 (52.16)	3,730 (50.71)	3,489 (51.69)	
Male	13,670 (48.24)	3,572 (47.33)	3,476 (47.84)	3,512 (49.29)	3,110 (48.31)	
Race, *n* (%)						< 0.0001
Mexican American	4,630 (8.28)	1,061 (7.69)	1,226 (8.59)	1,209 (8.64)	1,134 (8.12)	
Non-Hispanic Black	5,695 (10.91)	2040 (17.08)	1,480 (11.78)	1,275 (9.22)	900 (6.67)	
Non-Hispanic White	13,388 (69.99)	3,058 (64.41)	3,309 (68.08)	3,589 (71.57)	3,432 (74.80)	
Other Hispanic	2,225 (4.63)	536 (4.69)	589 (5.50)	578 (4.28)	522 (4.13)	
Other Race	2,123 (6.19)	398 (6.13)	523 (6.05)	591 (6.30)	611 (6.28)	
Marital status, *n* (%)						< 0.0001
Married/Living with a partner	16,876 (62.62)	3,862 (56.69)	4,228 (61.37)	4,493 (63.68)	4,293 (67.63)	
Never married	4,937 (18.36)	1,308 (19.60)	1,260 (18.52)	1,187 (17.49)	1,182 (18.08)	
Separated/Divorced/Widowed	6,238 (18.97)	1921 (23.66)	1,637 (20.09)	1,560 (18.78)	1,120 (14.24)	
No record	10 (0.04)	2 (0.04)	2 (0.02)	2 (0.06)	4 (0.05)	
Education level, *n* (%)						< 0.0001
<High School	7,393 (17.28)	2,490 (25.13)	2023 (19.08)	1,677 (15.25)	1,203 (11.17)	
High School	6,527 (23.47)	1842 (28.49)	1718 (24.94)	1,694 (23.66)	1,273 (17.74)	
>high school	14,141 (59.25)	2,761 (46.39)	3,386 (55.98)	3,871 (61.09)	4,123 (71.09)	
Poverty-to-income ratio, *n* (%)						< 0.0001
<1.3	8,116 (20.64)	2,577 (28.54)	2,171 (22.11)	1826 (17.54)	1,542 (15.85)	
1.3–3.5	9,773 (33.30)	2,589 (36.79)	2,542 (34.93)	2,536 (33.03)	2,106 (29.18)	
>3.5	8,183 (40.27)	1,414 (28.55)	1946 (37.76)	2,334 (43.11)	2,489 (49.47)	
No record	1989 (5.79)	513 (6.12)	468 (5.20)	546 (6.32)	462 (5.50)	
Hypertension, *n* (%)						< 0.0001
No	16,407 (62.38)	3,607 (56.37)	4,025 (60.15)	4,386 (63.09)	4,389 (68.70)	
Yes	11,654 (37.62)	3,486 (43.63)	3,102 (39.85)	2,856 (36.91)	2,210 (31.30)	
DM, *n* (%)						< 0.0001
No	22,583 (86.02)	5,477 (82.85)	5,679 (84.62)	5,928 (87.08)	5,499 (88.86)	
Yes	4,775 (12.63)	1,535 (16.39)	1,344 (14.55)	1,098 (11.39)	798 (9.00)	
No record	703 (1.35)	81 (0.76)	104 (0.84)	216 (1.53)	302 (2.15)	
CVD, *n* (%)						< 0.0001
No	24,902 (91.14)	5,903 (86.50)	6,281 (89.99)	6,581 (92.52)	6,137 (94.66)	
Yes	3,159 (8.86)	1,190 (13.50)	846 (10.01)	661 (7.48)	462 (5.34)	
Hyperlipidemia, *n* (%)						< 0.0001
No	7,733 (28.34)	1732 (24.32)	1856 (26.36)	2001 (28.15)	2,144 (33.68)	
Yes	20,328 (71.66)	5,361 (75.68)	5,271 (73.64)	5,241 (71.85)	4,455 (66.32)	
Cancer, *n* (%)						0.77
No	25,509 (90.71)	6,441 (90.71)	6,479 (90.28)	6,573 (90.86)	6,016 (90.94)	
Yes	2,552 (9.29)	652 (9.29)	648 (9.72)	669 (9.14)	583 (9.06)	
Total energy intake, kcal	2171.72 (9.43)	1437.04 (10.54)	1878.42 (12.50)	2350.13 (15.46)	2871.66 (22.00)	< 0.0001
Healthy eating index	50.86 (0.21)	43.92 (0.21)	48.85 (0.26)	51.76 (0.26)	57.56 (0.29)	< 0.0001
OBS	20.03 (0.12)	10.12 (0.04)	16.63 (0.03)	22.52 (0.03)	28.88 (0.04)	< 0.0001
Dietary OBS	16.15 (0.10)	6.98 (0.04)	12.95 (0.04)	18.59 (0.04)	24.22 (0.04)	< 0.0001
Lifestyle OBS	3.88 (0.03)	3.14 (0.03)	3.68 (0.03)	3.93 (0.04)	4.65 (0.04)	< 0.0001
Serum folate, nmol/L	44.84 (0.46)	39.96 (0.47)	43.61 (0.82)	45.43 (0.63)	49.52 (0.75)	< 0.0001
Red blood cells folate, nmol/L RBC	1218.78 (12.04)	1124.38 (11.82)	1210.70 (13.63)	1257.34 (15.50)	1273.79 (16.92)	< 0.0001
Vitamin B6, nmol/L	74.81 (1.95)	52.65 (1.94)	67.01 (2.73)	74.83 (1.93)	99.93 (4.27)	< 0.0001
Vitamin B12, pmol/L	458.45 (6.14)	426.60 (5.77)	458.24 (15.15)	476.27 (15.57)	468.85 (8.76)	< 0.001
HCY, μmol/L	8.81 (0.09)	9.72 (0.18)	8.95 (0.12)	8.66 (0.11)	7.98 (0.12)	< 0.0001
MMA, nmol/L	170.12 (1.89)	180.75 (3.52)	171.11 (2.63)	168.83 (3.29)	160.53 (2.49)	< 0.001

OBS, oxidative balance score; DM, diabetes mellitus; CVD, cardiovascular disease; HCY, homocysteine; MMA, methylmalonic acid. Data were presented as weighted percentages or means (95% confidence intervals). Q1–Q4: Quartiles of OBS (Q1 = lowest, Q4 = highest).

### 3.2 The association between OBS and methylation cycle biomarkers

Table 2 illustrates the results of the weighted linear regression analysis, which exposed a noteworthy link between the OBS and methylation cycle biomarkers. In the initial, unadjusted model, a significant positive correlation was observed between OBS and levels of serum folate (*β* = 9.56, 95% CI, 7.85–11.26, P for trend < 0.0001), RBC folate (*β* = 149.41, 95% CI, 117.30–181.53, P for trend < 0.0001), vitamin B6 (*β* = 47.28, 95% CI, 38.28–56.28, P for trend < 0.0001), and vitamin B12 (*β* = 42.25, 95% CI, 20.93–63.56, P for trend < 0.001). Conversely, a significant negative correlation was noted with HCY (*β* = −1.74, 95% CI, −2.15- -1.33, P for trend < 0.0001) and MMA (*β* = −20.21, 95% CI, −29.21- -11.22, P for trend < 0.001). Upon adjusting for age, sex, race, marital status, education, and poverty-income ratio in Model 1, the analysis showed that an elevated OBS was associated with higher concentrations of serum folate, RBC folate, vitamin B6, and vitamin B12, as well as lower levels of HCY. The significance of the association with MMA diminished. Further adjustment in Model 2, which included total energy intake, HEI, hypertension, CVD, diabetes, hyperlipidemia, and cancer, did not substantially alter the findings from Model 1. In summary, OBS was positively correlated with serum folate, RBC folate, vitamin B6, and vitamin B12, negatively correlated with HCY, and not significantly associated with MMA.

**Table 2:** Weighted linear regression showing the associations between OBS and methylation cycle biomarkers.

	Q1	Q2	*P*-value	Q3	*P*-value	Q4	*P*-value	P for trend
Serum folate								
Crude model	Ref	3.65 (1.85, 5.45)	<0.001	5.47 (3.93, 7.01)	<0.0001	9.56 (7.85, 11.26)	<0.0001	<0.0001
Model 1	Ref	2.82 (1.13, 4.50)	0.002	4.81 (3.28, 6.35)	<0.0001	8.93 (7.44, 10.43)	<0.0001	<0.0001
Model 2	Ref	3.84 (1.90, 5.78)	<0.001	6.57 (4.74, 8.39)	<0.0001	11.53 (9.28, 13.78)	<0.0001	<0.0001
RBC folate								
Crude model	Ref	86.32 (61.62, 111.02)	<0.0001	132.97 (104.30, 161.63)	<0.0001	149.41 (117.30, 181.53)	<0.0001	<0.0001
Model 1	Ref	66.19 (42.42, 89.96)	<0.0001	113.41 (82.84, 143.98)	<0.0001	129.82 (101.84, 157.80)	<0.0001	<0.0001
Model 2	Ref	71.59 (46.13, 97.05)	<0.0001	117.73 (84.98, 150.47)	<0.0001	137.99 (103.68, 172.30)	<0.0001	<0.0001
Vitamin B6								
Crude model	Ref	14.37 (8.00,20.73)	<0.0001	22.19 (17.92,26.45)	<0.0001	47.28 (38.28,56.28)	<0.0001	<0.0001
Model 1	Ref	11.31 (4.83, 17.78)	0.002	16.81 (12.13, 21.49)	<0.0001	39.99 (31.48, 48.51)	<0.0001	<0.0001
Model 2	Ref	10.76 (3.45, 18.08)	0.01	16.89 (9.93, 23.86)	<0.001	39.57 (28.95, 50.19)	<0.0001	<0.0001
Vitamin B12								
Crude model	Ref	31.64 (−3.72,67.00)	0.08	49.66 (14.66,84.67)	0.01	42.25 (20.93, 63.56)	<0.001	<0.001
Model 1	Ref	30.04 (−7.60, 67.69)	0.11	49.8 (12.27, 87.32)	0.01	51.6 (26.09, 77.12)	<0.001	<0.001
Model 2	Ref	42.6 (−4.37, 89.56)	0.07	75.03 (29.49, 120.57)	0.01	88.98 (40.02, 137.93)	0.004	<0.001
HCY								
Crude model	Ref	−0.77 (−1.14, −0.40)	<0.001	−1.05 (−1.43, −0.68)	<0.0001	−1.74 (−2.15, −1.33)	<0.0001	<0.0001
Model 1	Ref	−0.6 (−0.96, −0.24)	0.003	−0.83 (−1.23, −0.44)	<0.001	−1.31 (−1.65, −0.98)	<0.0001	<0.0001
Model 2	Ref	−0.69 (−1.12, −0.25)	0.01	−1.06 (−1.56, −0.55)	0.003	−1.65 (−2.14, −1.15)	<0.001	<0.0001
MMA								
Crude model	Ref	−9.64 (−17.49, −1.79)	0.02	−11.92 (−22.08, −1.76)	0.02	−20.21 (−29.21, −11.22)	<0.0001	<0.001
Model 1	Ref	−8.7 (−16.12, −1.29)	0.02	−9.57 (−19.77, 0.63)	0.06	−14.07 (−22.50, −5.64)	0.003	0.01
Model 2	Ref	−8.28 (−16.70, 0.15)	0.05	−9.00 (−21.98, 3.99)	0.15	−13.5 (−27.13, 0.12)	0.05	0.07

Crudel model: unadjusted model; Model 1: adjusted for age, sex, race, marital status, education, and poverty-income ratio; Model 2: additionally adjusted for total energy intake, HEI, hypertension, CVD, diabetes, hyperlipidemia, and cancer. Ref: Reference group (quartile Q1, lowest OBS).

### 3.3 Relationship of dietary OBS and lifestyle OBS with methylation cycle biomarkers

Table 3 presents the linear regression outcomes examining the association between dietary and lifestyle components of the OBS and markers of the methylation cycle. The unadjusted analysis indicated that an increased dietary OBS was associated with elevated levels of serum folate (*β* = 7.75, 95% CI, 5.95–9.56, P for trend < 0.0001), RBC folate (*β* = 161.34, 95% CI, 128.74–193.93, P for trend < 0.0001), vitamin B6 (*β* = 41.30, 95% CI, 33.22–49.38, P for trend < 0.0001), and vitamin B12 (*β* = 39.13, 95% CI, 10.29–67.97, P for trend = 0.003), as well as reduced levels of HCY (*β* = −1.66, 95% CI, −2.13 to −1.19, P for trend < 0.0001) and MMA (*β* = −17.78, 95% CI, −26.35 to −9.21, P for trend < 0.001). However, after adjusting for various covariates in Model 1, the dietary OBS was found to have statistically significant correlations only with serum folate, RBC folate, vitamin B6, vitamin B12, and HCY. For the lifestyle OBS, the unadjusted model suggested that higher scores were linked to higher concentrations of serum folate, vitamin B6, and vitamin B12, along with lower levels of HCY and MMA. Upon adjustment in Model 1, the lifestyle OBS exhibited positive associations with serum folate, vitamin B6, and vitamin B12, and a negative association with HCY. In conclusion, both dietary and lifestyle aspects of OBS demonstrated correlations with the methylation cycle.

**Table 3:** Weighted linear regression showing the associations between dietary OBS, lifestyle OBS, and methylation cycle biomarkers.

	Crude model		Model 1		Crude model		Model 1	
	*β* (95%CI)	*P*-value	*β* (95%CI)	*P*-value	*β* (95%CI)	*P*-value	*β* (95%CI)	*P*-value
	Dietary OBS				Lifestyle OBS			
Serum folate								
Q1	Ref		Ref		Ref		Ref	
Q2	2.62 (1.06, 4.17)	0.001	2.3 (0.61, 3.98)	0.01	2.66 (1.20, 4.11)	<0.001	2.76 (1.32, 4.19)	<0.001
Q3	5.15 (3.12, 7.19)	<0.0001	5.67 (3.41, 7.92)	<0.0001	5.65 (3.81, 7.49)	<0.0001	6.03 (4.24, 7.81)	<0.0001
Q4	7.75 (5.95, 9.56)	<0.0001	8.93 (6.78, 11.08)	<0.0001	10.03 (8.25, 11.81)	<0.0001	8.74 (6.89, 10.59)	<0.0001
P for trend		<0.0001		<0.0001		<0.0001		<0.0001
RBC folate								
Q1	Ref		Ref		Ref		Ref	
Q2	84.22 (61.23, 107.21)	<0.0001	69.25 (41.30, 97.19)	<0.0001	−1.21 (−28.37, 25.96)	0.93	6.03 (−20.47, 32.53)	0.65
Q3	135.26 (101.04, 169.49)	<0.0001	126.11 (88.72, 163.49)	<0.0001	4.99 (−22.81, 32.79)	0.72	23.25 (−6.44, 52.94)	0.12
Q4	161.34 (128.74, 193.93)	<0.0001	157.07 (121.90, 192.24)	<0.0001	29.81 (−6.27, 65.90)	0.1	29.89 (−5.79, 65.56)	0.1
P for trend		<0.0001		<0.0001		0.13		0.07
Vitamin B6								
Q1	Ref		Ref		Ref		Ref	
Q2	14.76 (7.81, 21.72)	<0.001	10.45 (2.01, 18.88)	0.02	19.86 (12.00, 27.72)	<0.0001	12.99 (5.49, 20.48)	0.005
Q3	23.64 (17.77, 29.52)	<0.0001	17.51 (9.06, 25.97)	0.002	23.08 (17.73, 28.43)	<0.0001	14.27 (8.47, 20.07)	<0.001
Q4	41.30 (33.22, 49.38)	<0.0001	33.40 (23.57, 43.24)	<0.0001	51.35 (39.76, 62.95)	<0.0001	33.88 (21.89, 45.88)	<0.001
P for trend		<0.0001		<0.0001		<0.0001		<0.0001
Vitamin B12								
Q1	Ref		Ref		Ref		Ref	
Q2	33.97 (−16.08, 84.02)	0.18	43.62 (−18.79, 106.03)	0.14	4.16 (−17.81, 26.13)	0.7	9.27 (−16.22, 34.75)	0.42
Q3	45.88 (4.14, 87.62)	0.03	69.21 (22.00, 116.42)	0.01	26.65 (−12.60, 65.89)	0.18	33.54 (−10.38, 77.45)	0.11
Q4	39.13 (10.29, 67.97)	0.01	81.53 (27.60, 135.45)	0.01	39.3 (12.97, 65.62)	0.005	46.16 (11.24, 81.08)	0.02
P for trend		0.003		0.001		0.003		0.01
HCY								
Q1	Ref		Ref		Ref		Ref	
Q2	−0.62 (−1.03, −0.20)	0.01	−0.6 (−1.07, −0.14)	0.02	−0.36 (−0.80, 0.08)	0.10	−0.28 (−0.73, 0.16)	0.16
Q3	−1.09 (−1.51, −0.68)	<0.0001	−1.1 (−1.62, −0.58)	0.003	−0.65 (−0.92, −0.38)	<0.0001	−0.39 (−0.74, −0.04)	0.03
Q4	−1.66 (−2.13, −1.19)	<0.0001	−1.63 (−2.22, −1.03)	<0.001	−0.98 (−1.36, −0.59)	<0.0001	−0.69 (−1.14, −0.24)	0.01
P for trend		<0.0001		<0.001		<0.0001		0.01
MMA								
Q1	Ref		Ref		Ref		Ref	
Q2	−8.53 (−16.40, −0.67)	0.03	−7.18 (−16.07, 1.72)	0.1	−7.77 (−14.71, −0.83)	0.03	−2.84 (−11.35, 5.67)	0.46
Q3	−11.57 (−21.62, −1.52)	0.03	−8.17 (−20.22, 3.89)	0.15	−4.96 (−12.47, 2.54)	0.19	1.96 (−7.41, 11.33)	0.64
Q4	−17.78 (−26.35, −9.21)	<0.001	−11.81 (−24.44, 0.82)	0.06	−13.23 (−21.14, −5.33)	0.002	−4.05 (−13.76, 5.66)	0.36
P for trend		<0.001		0.09		0.005		0.63

Crudel model: unadjusted model; Model 1: Adjusted for age, sex, race, marital status, education, poverty-income ratio, total energy intake, HEI, hypertension, CVD, diabetes, hyperlipidemia, and cancer. Ref: Reference group (quartile Q1, lowest OBS).

### 3.4 Stratified analyses of OBS and methylation cycle biomarkers

Table 4 displayed the results of stratified analyses examining the relationship between OBS and serum folate, RBC folate, and vitamin B6. The findings indicated a strong positive correlation between OBS and these biomarkers, with this positive association remaining consistent across most stratification factors. Supplementary Table S2 presented the results of stratified analyses for OBS with vitamin B12, HCY, and MMA, demonstrating a strong negative correlation with HCY and a strong positive correlation with vitamin B12. The associations persisted across most stratification factors, including age, sex, race, education, marital status, poverty, hypertension, diabetes, hyperlipidemia, CVD, and cancer. Notably, while a negative correlation between OBS and MMA was observed in most stratification factors, it was only present in the elderly population (≥ 60 years old) when stratified by age.

**Table 4:** Stratified analysis of OBS and methylation cycle biomarkers.

Character	Serum folate			RBC folate			Vitamin B6		
	*β* (95%CI)	*p*-value	p for interaction	*β* (95%CI)	*p*-value	p for interaction	*β* (95%CI)	*p*-value	p for interaction
Age			0.55			0.62			0.36
20–39	0.56 (0.43, 0.69)	<0.0001		10.82 (8.92, 12.73)	<0.0001		2.36 (1.79, 2.93)	<0.0001	
40–59	0.6 (0.49, 0.72)	<0.0001		9.45 (7.17, 11.73)	<0.0001		2.89 (2.10, 3.67)	<0.0001	
≥60	0.68 (0.47, 0.90)	<0.0001		9.17 (4.71, 13.64)	<0.001		2.75 (2.11, 3.38)	<0.0001	
Sex			< 0.001			0.01			0.17
Female	0.69 (0.56, 0.81)	<0.0001		10.35 (8.03, 12.68)	<0.0001		2.84 (2.19, 3.48)	<0.0001	
Male	0.38 (0.25, 0.51)	<0.0001		6.74 (4.56, 8.92)	<0.0001		2.36 (1.83, 2.89)	<0.0001	
Race			0.03			0.46			0.02
Mexican American	0.18 (−0.04, 0.41)	0.11		4.52 (1.39, 7.66)	0.01		1.59 (0.95, 2.22)	<0.0001	
Non-Hispanic White	0.51 (0.39, 0.63)	<0.0001		7.42 (4.99, 9.86)	<0.0001		2.8 (2.20, 3.40)	<0.0001	
Non-Hispanic Black	0.33 (0.20, 0.45)	<0.0001		5.53 (3.46, 7.60)	<0.0001		1.67 (1.10, 2.24)	<0.0001	
Other Hispanic	0.55 (0.38, 0.73)	<0.0001		5.85 (2.63, 9.08)	<0.001		2.2 (1.58, 2.81)	<0.0001	
Other Race	0.56 (0.28, 0.84)	<0.001		6.65 (1.57, 11.73)	0.01		1.89 (0.42, 3.35)	0.01	
Marital			0.14			0.5			0.9
Married/Living with a partner	0.55 (0.44, 0.67)	<0.0001		8.7 (6.67, 10.73)	<0.0001		2.58 (1.96, 3.21)	<0.0001	
Never married	0.41 (0.21, 0.61)	<0.001		7.51 (4.76, 10.26)	<0.0001		2.7 (1.92, 3.48)	<0.0001	
Separated/Divorced/Widowed	0.66 (0.51, 0.82)	<0.0001		9.68 (6.73, 12.64)	<0.0001		2.45 (1.63, 3.27)	<0.0001	
Education			0.37			0.08			0.01
<High School	0.39 (0.20, 0.59)	<0.001		7.08 (2.84, 11.33)	0.001		1.64 (1.18, 2.11)	<0.0001	
High School	0.61 (0.40, 0.82)	<0.0001		11.89 (8.54, 15.24)	<0.0001		1.72 (1.07, 2.36)	<0.0001	
>High School	0.51 (0.37, 0.65)	<0.0001		6.99 (4.35, 9.63)	<0.0001		2.89 (2.20, 3.58)	<0.0001	
Poverty-to-income ratio			0.21			0.44			0.02
<1.3	0.49 (0.35, 0.63)	<0.0001		5.69 (3.22, 8.17)	<0.0001		1.69 (1.39, 2.00)	<0.0001	
1.3–3.5	0.6 (0.43, 0.76)	<0.0001		8.96 (5.83, 12.10)	<0.0001		1.83 (1.26, 2.39)	<0.0001	
>3.5	0.43 (0.23, 0.64)	<0.0001		7.17 (4.11, 10.22)	<0.0001		3.12 (2.19, 4.04)	<0.0001	
No record	0.21 (−0.16, 0.57)	0.26		6.98 (1.31, 12.66)	0.02		2.75 (1.12, 4.38)	0.002	
Hypertension			0.53			0.64			0.31
No	0.61 (0.51, 0.71)	<0.0001		10.47 (8.87, 12.08)	<0.0001		2.72 (2.07, 3.38)	<0.0001	
Yes	0.55 (0.38, 0.72)	<0.0001		9.67 (6.21, 13.13)	<0.0001		2.34 (1.89, 2.80)	<0.0001	
DM			0.71			0.64			0.06
No	0.58 (0.48, 0.68)	<0.0001		9.55 (7.55, 11.56)	<0.0001		2.69 (2.14, 3.24)	<0.0001	
Yes	0.48 (0.19, 0.76)	0.001		8.01 (3.20, 12.81)	0.001		1.87 (1.35, 2.38)	<0.0001	
No record	0.56 (−0.12, 1.24)	0.1		14.62 (0.70, 28.54)	0.04		2.15 (0.28, 4.01)	0.03	
Hyperlipidemia			0.73			0.9			0.22
No	0.52 (0.33, 0.71)	<0.0001		9.23 (6.92, 11.53)	<0.0001		3.05 (2.09, 4.02)	<0.0001	
Yes	0.56 (0.45, 0.67)	<0.0001		9.4 (7.27, 11.52)	<0.0001		2.43 (1.96, 2.91)	<0.0001	
CVD			0.04			0.06			0.07
No	0.56 (0.47, 0.65)	<0.0001		9.21 (7.39, 11.04)	<0.0001		2.66 (2.16, 3.17)	<0.0001	
Yes	0.89 (0.56, 1.22)	<0.0001		15.65 (8.94, 22.35)	<0.0001		1.92 (1.25, 2.59)	<0.0001	
Cancer			0.48			0.69			0.77
No	0.53 (0.43, 0.62)	<0.0001		8.71 (6.87, 10.54)	<0.0001		2.63 (2.14, 3.11)	<0.0001	
Yes	0.64 (0.33, 0.94)	<0.001		7.4 (0.77, 14.03)	0.03		2.48 (1.37, 3.59)	<0.0001	

### 3.5 Restricted cubic spline analysis

**Figure 1:**
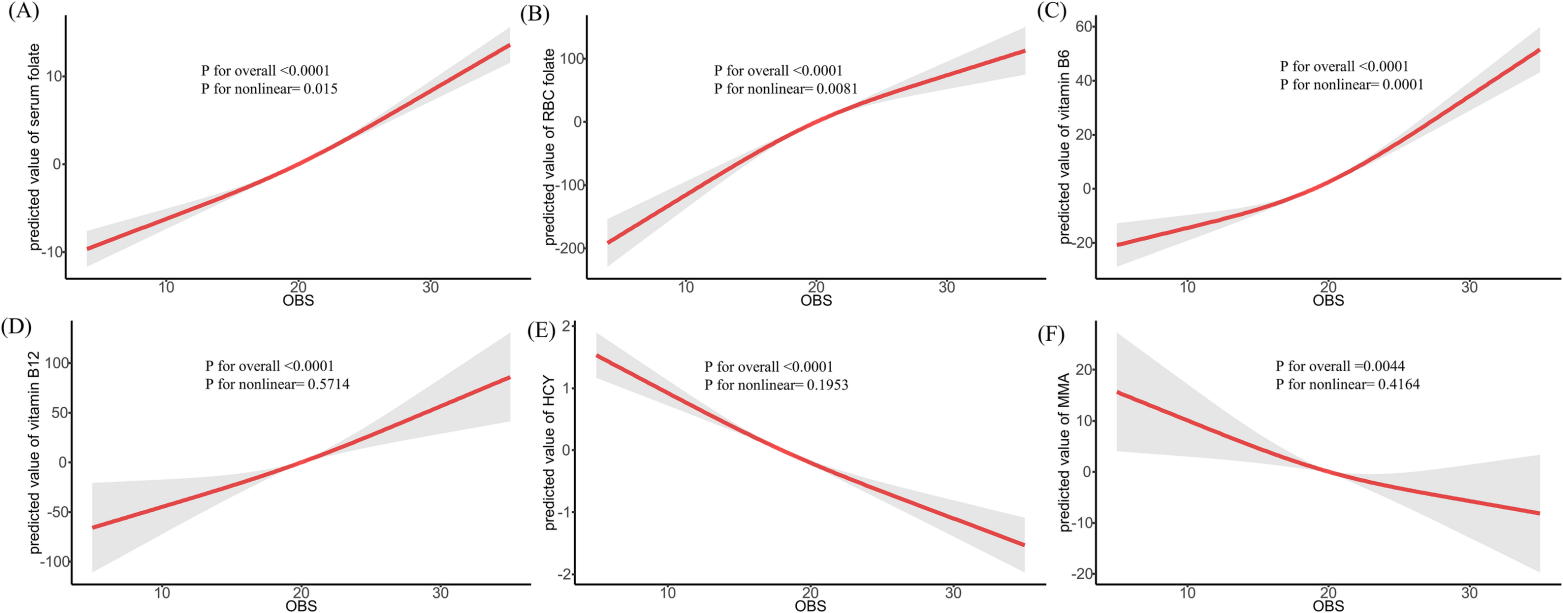
Restricted cubic spline regression between OBS and methylation cycle biomarkers. *Adjusted for age, race, education, marital status, PIR, HEI, total energy intake, hypertension, DM, CVD, hyperlipidemia, and cancer; OBS: oxidative balance score.

The RCS regression analysis, which accounted for various confounders such as age, race, education, marital status, PIR, HEI, total energy intake, hypertension, DM, CVD, hyperlipidemia, and cancer, was conducted. As depicted in Figure 1, there was a significant positive trend observed between increasing OBS and the levels of serum folate, RBC folate, vitamin B6, and vitamin B12, while levels of HCY and MMA showed a decline. Furthermore, Supplementary Figure S1 showed that a higher dietary OBS correlated with elevated levels of serum folate, RBC folate, vitamin B6, and vitamin B12, and reduced levels of HCY and MMA. Supplementary Figure S2 illustrated that the lifestyle OBS exhibited positive associations with serum folate, vitamin B6, vitamin B12, and HCY, a negative association with RBC folate, and an inverse U-shaped relationship with MMA.

## 4 Discussion

The present study, utilizing a large, representative sample from NHANES, has provided comprehensive insights into the association between the OBS and biomarkers of the methylation cycle among US adults. Our findings reveal a consistent positive correlation between OBS and serum folate, RBC folate, vitamin B6, and vitamin B12 levels, suggesting that a higher OBS is indicative of a more optimal one-carbon metabolism. Conversely, a negative correlation was observed between OBS and HCY levels, and a complex relationship with MMA was noted, primarily in the elderly population. Both dietary OBS and lifestyle OBS showed significant associations with the methylation cycle biomarkers, underlining the multifaceted nature of their interaction. RCS regression further confirmed these trends, highlighting the robustness of our findings.

The positive correlation between the OBS and the levels of folate and vitamins B6 and B12 is rooted deeply in the interconnected pathways of one-carbon metabolism and antioxidant defense. The central role of these nutrients in the methylation cycle, particularly in the conversion of homocysteine to methionine and the subsequent synthesis of S-adenosylmethionine (SAM), underscores their vital importance in biological processes (16). SAM, acting as the universal methyl donor, is a linchpin in DNA synthesis, repair, and epigenetic regulation, which are fundamental to cellular function and health (17). The tight coupling between the folate cycle and the methylation cycle ensures a continuous supply of methyl groups for these critical processes. Folate and vitamins B6 and B12 serve as essential cofactors and regulators in this cycle, facilitating the efficient conversion of homocysteine to methionine and the regeneration of tetrahydrofolate (THF) from 5,10-methylene-THF (18). This regeneration step maintains the availability of one-carbon units for the synthesis of thymidylate and purines, essential components of DNA and RNA, respectively (19).

A higher OBS, indicative of a more robust antioxidant defense system, can significantly impact one-carbon metabolism by protecting these critical nutrients and their cofactors from oxidative damage. Oxidative stress, characterized by an imbalance between ROS production and antioxidant defenses, can lead to the peroxidation of lipids, proteins, and DNA, as well as the inactivation of enzymes and cofactors involved in one-carbon metabolism (20). The resulting oxidative damage can compromise the integrity of the methylation cycle, potentially leading to reduced DNA synthesis and repair, aberrant DNA methylation patterns, and other epigenetic alterations (21). By maintaining a high OBS, individuals can mitigate such oxidative damage, preserving the optimal function of enzymes and cofactors in one-carbon metabolism. This preservation ensures the efficient synthesis and recycling of folate, vitamins B6 and B12, and other critical components of the methylation cycle. Consequently, a higher OBS supports optimal DNA synthesis, repair, and epigenetic regulation, which are paramount for maintaining genomic stability and preventing diseases associated with these processes, including cardiovascular diseases, neurological disorders, and cancer (22).

The findings of this study, revealing a positive correlation between OBS and folate and vitamin B6/12 levels, have significant implications for our understanding of the interplay between oxidative stress, one-carbon metabolism, and health. We suggest that strategies aimed at improving OBS, such as dietary modifications to increase antioxidant intake or lifestyle changes to reduce oxidative stress, could potentially enhance one-carbon metabolism and, by extension, DNA synthesis, repair, and epigenetic regulation. For individuals with compromised one-carbon metabolism, such as those with cardiovascular disease or neurological disorders, optimizing OBS might represent a promising avenue for intervention. By improving the antioxidant defense system, it is possible to reduce oxidative damage to critical nutrients and cofactors, thereby supporting the methylation cycle and potentially mitigating disease progression. In summary, the positive correlations between OBS and folate and vitamin B6/12 levels highlight the synergistic relationship between antioxidant defense and one-carbon metabolism. This relationship is crucial for maintaining optimal biological processes and genomic stability, with potential implications for the prevention and management of a wide range of diseases.

The inverse relationship between the OBS and HCY levels was multifaceted, involving intricate interactions between OS, antioxidant defense, and the metabolic pathways of HCY. OS, characterized by an imbalance between pro-oxidants and antioxidants, can disrupt the delicate equilibrium of HCY metabolism by affecting the transsulfuration and remethylation pathways (23). These pathways are crucial for the conversion of HCY back to methionine or to cystathionine, which is further metabolized to cysteine, a component of glutathione, a potent antioxidant (24). A higher OBS, indicative of better antioxidant status, can potentially enhance the transsulfuration and remethylation pathways. This enhancement is facilitated by the adequate intake of specific nutrients that contribute to a higher OBS, notably folate and vitamins B6 and B12. Folate and vitamin B12 are essential for the remethylation of HCY to methionine, while vitamin B6 is a cofactor for the transsulfuration pathway that converts HCY to cystathionine (25). Folate and vitamin B12 act as methyl donors in the remethylation pathway, converting HCY to methionine in a reaction that also generates THF from 5-methyl-THF (16). This process is catalyzed by methionine synthase, an enzyme that requires vitamin B12 as a cofactor. A higher OBS can support the integrity of this pathway by protecting vitamin B12 from oxidative damage, ensuring its availability for the methylation reaction (26). Vitamin B6, as a cofactor for the enzyme cystathionine *β*-synthase, plays a key role in the transsulfuration pathway and this pathway is an alternative route for HCY metabolism that does not require vitamin B12 (27). Instead, it produces cysteine, which is a precursor for the synthesis of glutathione, a key antioxidant in the cell. A higher OBS, by promoting vitamin B6 intake and activity, can enhance the transsulfuration pathway, facilitating the conversion of HCY to cysteine and, by extension, the synthesis of glutathione (28). A higher OBS, by improving the efficiency of the transsulfuration and remethylation pathways, can reduce oxidative stress and HCY accumulation. This reduction in oxidative stress further supports these metabolic pathways, creating a positive feedback loop that enhances HCY metabolism. Moreover, the enhanced synthesis of glutathione, a direct result of the transsulfuration pathway, strengthens the antioxidant defense system, contributing to a lower OBS.

The negative correlation between OBS and HCY highlights the potential of dietary and lifestyle modifications aimed at improving OBS as a strategy for mitigating HCY accumulation and, by extension, reducing cardiovascular risk. Clinical interventions focusing on optimizing OBS through a balanced diet rich in antioxidant-rich foods and a healthy lifestyle could contribute to the prevention and management of cardiovascular diseases associated with hyperhomocysteinemia (29).

The intricate relationship between the OBS and MMA was particularly pronounced among the elderly population, showcasing the multifaceted nature of aging on one-carbon metabolism and vitamin B12 status. MMA, a sensitive indicator of vitamin B12 functionality and health of the methylation cycle portrays a complex relationship with OBS in seniors, reflecting age-related changes in vitamin B12 absorption, transport, and metabolism, as well as the cumulative effects of oxidative stress on MMA metabolism enzymes (23). With advancing age, significant alterations in vitamin B12 absorption and metabolism occur, impacting MMA levels in the elderly (9). Decreased gastric acid secretion impairs vitamin B12 absorption, potentially elevating MMA levels despite adequate intake (30). Moreover, vitamin B12 transport from the intestine to target organs is affected by age-related reductions in protein synthesis, decreasing MMA clearance efficiency (31). The relationship between OBS and MMA is complicated by oxidative stress’s impact on the methylation cycle. OS damages MMA metabolism enzymes, leading to its accumulation, a feature more pronounced in older adults experiencing chronic low-grade inflammation and oxidative stress (32). Long-term oxidative stress leads to oxidative damage to MAA metabolism enzymes, reducing elderly individuals’ capacity to metabolize MMA and complicating the OBS-MMA relationship. Age-related decline in renal function affects MMA clearance. Reduced kidney function can elevate MMA levels, even under normal or elevated vitamin B12 status (33, 34). Decreased renal MMA clearance might obscure the effects of improved OBS on MMA levels. The subtle OBS-MMA relationship in the elderly highlights the significance of considering age-related alterations in vitamin B12 metabolism, renal function, and oxidative stress. Tailored strategies to enhance OBS through dietary and lifestyle modifications for the elderly are crucial for effectively diminishing MMA accumulation and supporting methylation cycle functionality.

A key strength of this study was its utilization of the NHANES database, which provided a rich, nationally representative dataset. This allowed for the generalization of our findings to the broader US population. Additionally, the comprehensive nature of OBS, encompassing a wide range of dietary and lifestyle factors, provided a more holistic assessment of oxidative balance than studies focusing on a single factor. The use of advanced statistical methods, including weighted linear regression and RCS analysis, further enhanced the robustness of our findings.

This study has several limitations. Firstly, the cross-sectional design precluded any inference of causality, and the observed associations may be subject to unmeasured confounding factors. Secondly, the OBS was developed based on established knowledge linking oxidative stress with nutrients and lifestyle factors, and while it has been used in previous studies, there may be other factors that influence oxidative balance that are not captured by the current OBS components. Thirdly, the selection of specific years for biomarker data was constrained by the availability within the NHANES database. For example, the measurement of vitamin B12 and MMA was discontinued after 2014, limiting our data to that time frame. This selective inclusion of years may affect the generalizability of our findings, and future research could explore methods to integrate more recent data while accounting for methodological changes. Fourthly, the assessment of dietary intake relies on self-reported data, which is susceptible to recall bias and may not accurately reflect actual consumption patterns. Lastly, while our findings suggest a relationship between OBS and methylation cycle biomarkers, the biological mechanisms underlying this association require further elucidation in prospective studies or experimental designs.

## 5 Conclusion

In conclusion, our study using NHANES data reveals a significant association between the OBS and methylation cycle biomarkers in US adults. A higher OBS correlates positively with optimal levels of folate and vitamins B6 and B12, and negatively with homocysteine, while the relationship with MMA is complex, particularly among older adults. These findings underscore the importance of maintaining a robust antioxidant defense system for healthy one-carbon metabolism and suggest potential strategies for disease prevention through lifestyle and dietary modifications. Further research is needed to explore the underlying mechanisms and validate these associations in different populations.

## Data Availability

Publicly available datasets were analyzed in this study. This data can be found at: https://www.cdc.gov/nchs/nhanes/index.html.

## Footnotes

^1^ https://www.cdc.gov/nchs/data_access/restrictions.htm
