# Supplementary material for: Association between oxidative balance score and methylation cycle biomarkers in US adults: insights from the national health and nutrition examination survey
Source: Front Nutr. 2025 Apr 28;12:1526025. doi: 10.3389/fnut.2025.1526025 (PMC12068862; doi:10.3389/fnut.2025.1526025)
Supplement: Supplementary file 1 [file Table_1.docx]

Supplementary Material

Association between Oxidative Balance Score and Methylation Cycle Biomarkers: Insights from the National Health and Nutrition Examination Survey

Xuna Liu ^1^, Yiwen Wang ^2,^*

*** Correspondence:**Yiwen Wang
wangyiwenmoon@stu.xjtu.edu.cn

# Supplementary Tables and Figures

## Supplementary Tables

Table S1. Components of the oxidative balance score (1)

| OBS components | Property | Female | | | Male | | |
| --- | --- | --- | --- | --- | --- | --- | --- |
|  |  | 0 | 1 | 2 | 0 | 1 | 2 |
| Lifestyle OBS components |  |  |  |  |  |  |  |
| Physical activity (MET-minute/week) | A | <270.00 | 270.00-845.71 | ≥845.71 | <417.86 | 417.86-1135.71 | ≥1135.71 |
| Alcohol (g/d) | P | ≥15 | 0-15 | None | ≥30 | 0-30 | None |
| Cotinine (ng/mL) | P | ≥0.035 | 0.035-0.172 | <0.172 | ≥0.038 | 0.038-1.13 | <1.13 |
| Body mass index (kg/m2) | P | ≥23.74 | 23.74-28.64 | <28.64 | ≥25.54 | 25.54-29.17 | <29.17 |
| Dietary OBS components |  |  |  |  |  |  |  |
| Dietary fiber (g/d) | A | <10.10 | 10.10-16.31 | ≥16.31 | <12.56 | 12.56-19.70 | ≥19.70 |
| Carotene (RE/d) | A | <98.08 | 98.08-383.50 | ≥383.50 | <98.83 | 98.83-306.25 | ≥306.25 |
| Riboflavin (mg/d) | A | <1.34 | 1.34-2.02 | ≥2.02 | <1.79 | 1.79-2.69 | ≥2.69 |
| Niacin (mg/d) | A | <14.52 | 14.52-21.86 | ≥21.86 | <20.65 | 20.65-29.75 | ≥29.75 |
| Vitamin B6 (mg/d) | A | <1.13 | 1.13-1.77 | ≥1.77 | <1.59 | 1.59-2.40 | ≥2.40 |
| Total folate (mcg/d) | A | <251.00 | 251.00-388.96 | ≥388.96 | <316.00 | 316.00-492.00 | ≥492.00 |
| Vitamin B12 (mcg/d) | A | <2.22 | 2.22-4.22 | ≥4.22 | <3.36 | 3.36-6.20 | ≥6.20 |
| Vitamin C (mg/d) | A | <38.01 | 38.01-98.49 | ≥98.49 | <42.44 | 42.44-113.21 | ≥113.21 |
| Vitamin E (ATE) (mg/d) | A | <4.53 | 4.53-7.52 | ≥7.52 | <5.82 | 5.82-9.42 | ≥9.42 |
| Calcium (mg/d) | A | <499.24 | 499.24-849.00 | ≥849.00 | <646.00 | 646.00-1072.00 | ≥1072.00 |
| Magnesium (mg/d) | A | <187.00 | 187.00-283.43 | ≥283.43 | <257.00 | 257.00-361.28 | ≥361.28 |
| Zinc (mg/d) | A | <6.73 | 6.73-10.75 | ≥10.75 | <9.75 | 9.75-15.10 | ≥15.10 |
| Copper (mg/d) | A | <0.85 | 0.85-1.28 | ≥1.28 | <1.12 | 1.12-1.57 | ≥1.57 |
| Selenium (mcg/d) | A | <67.79 | 67.79-99.50 | ≥99.50 | <94.94 | 94.94-141.80 | ≥141.80 |
| Total fat (g/d) | P | ≥50.98 | 50.98-75.79 | <75.79 | ≥69.83 | 69.83-107.43 | <107.43 |
| Iron (mg/d) | P | ≥9.65 | 9.65-14.32 | <14.32 | ≥12.88 | 12.88-19.17 | <19.17 |

OBS: oxidative balance score; A: antioxidant; P: prooxidant; RE: retinol equivalent; ATE: alpha-tocopherol equivalent; MET: metabolic equivalent.

Reference

1. Zhang W, Peng S-F, Chen L, Chen H-M, Cheng X-E, Tang Y-H. Association between the Oxidative Balance Score and Telomere Length from the National Health and Nutrition Examination Survey 1999-2002. *Oxid Med Cell Longev* (2022) 2022:1345071. doi: 10.1155/2022/1345071

Table S2. Stratified analysis of OBS and methylation cycle biomarkers.

|  | **Vitamin B12** | | |  | **HCY** | | |  | **MMA** | | |
| --- | --- | --- | --- | --- | --- | --- | --- | --- | --- | --- | --- |
| Character | β (95%CI) | p value | p for interaction |  | β (95%CI) | p value | p for interaction |  | β (95%CI) | p value | p for interaction |
| Age |  |  | 0.53 |  |  |  | 0.01 |  |  |  | < 0.0001 |
| 20-39 | 3.4(2.08,4.72) | <0.0001 |  |  | -0.05(-0.08,-0.03) | <0.001 |  |  | -0.11(-0.53,0.31) | 0.6 |  |
| 40-59 | 1.51(-1.18,4.21) | 0.26 |  |  | -0.09(-0.14,-0.05) | <0.0001 |  |  | -0.56(-1.39,0.27) | 0.18 |  |
| ≥60 | 3.02(-0.84,6.88) | 0.12 |  |  | -0.12(-0.14,-0.09) | <0.0001 |  |  | -2.28(-3.12,-1.44) | <0.0001 |  |
| Sex |  |  | 0.03 |  |  |  | 0.6 |  |  |  | 0.68 |
| Female | 0.7(-1.57,2.97) | 0.53 |  |  | -0.09(-0.12,-0.06) | <0.0001 |  |  | -0.96(-1.60,-0.33) | 0.004 |  |
| Male | 3.95(2.38,5.51) | <0.0001 |  |  | -0.1(-0.13,-0.07) | <0.0001 |  |  | -1.17(-1.89,-0.46) | 0.002 |  |
| Race |  |  | 0.25 |  |  |  | 0.07 |  |  |  | 0.62 |
| Mexican American | 1.93(-2.41,6.26) | 0.37 |  |  | -0.05(-0.08,-0.03) | <0.001 |  |  | -1.24(-2.11,-0.38) | 0.01 |  |
| Non-Hispanic White | 1.77(0.11,3.44) | 0.04 |  |  | -0.11(-0.13,-0.08) | <0.0001 |  |  | -1.15(-1.66,-0.64) | <0.0001 |  |
| Non-Hispanic Black | 4.25(2.22,6.29) | <0.001 |  |  | -0.07(-0.13,-0.02) | 0.01 |  |  | -0.89(-1.61,-0.18) | 0.02 |  |
| Other Hispanic | 2.46(-2.62,7.54) | 0.33 |  |  | -0.08(-0.13,-0.03) | 0.004 |  |  | -1.77(-3.22,-0.33) | 0.02 |  |
| Other Race | 5.31(2.44,8.18) | <0.001 |  |  | -0.06(-0.13,0.00) | 0.05 |  |  | -1.89(-3.87,0.09) | 0.06 |  |
| Marital |  |  | 0.04 |  |  |  | 0.21 |  |  |  | 0.06 |
| Married/Living with partner | 0.94(-0.98,2.86) | 0.32 |  |  | -0.08(-0.11,-0.06) | <0.0001 |  |  | -0.71(-1.14,-0.28) | 0.002 |  |
| Never married | 3.81(2.13,5.49) | <0.0001 |  |  | -0.07(-0.12,-0.03) | 0.002 |  |  | -0.59(-1.58,0.41) | 0.24 |  |
| Separated/Divorced/Widowed | 5.14(1.11,9.18) | 0.01 |  |  | -0.12(-0.18,-0.07) | <0.0001 |  |  | -2.15(-3.35,-0.94) | <0.001 |  |
| Education |  |  | 0.15 |  |  |  | 0.11 |  |  |  | 0.07 |
| <High School | 4.76(1.36,8.16) | 0.01 |  |  | -0.12(-0.16,-0.08) | <0.0001 |  |  | -1.91(-2.77,-1.06) | <0.0001 |  |
| High School | 4.65(0.67,8.63) | 0.02 |  |  | -0.07(-0.11,-0.03) | 0.002 |  |  | -0.74(-1.53,0.06) | 0.07 |  |
| >High School | 1.28(-0.84,3.41) | 0.23 |  |  | -0.08(-0.11,-0.06) | <0.0001 |  |  | -0.86(-1.40,-0.33) | 0.002 |  |
| Poverty-to-income ratio |  |  | 0.16 |  |  |  | 0.94 |  |  |  | 0.19 |
| <1.3 | 3.29(1.92,4.65) | <0.0001 |  |  | -0.09(-0.14,-0.04) | <0.001 |  |  | -1.42(-2.35,-0.49) | 0.004 |  |
| 1.3-3.5 | 4.04(1.82,6.26) | <0.001 |  |  | -0.1(-0.13,-0.06) | <0.0001 |  |  | -1.15(-1.85,-0.46) | 0.002 |  |
| >3.5 | -0.53(-4.38,3.32) | 0.78 |  |  | -0.09(-0.12,-0.06) | <0.0001 |  |  | -0.44(-1.11,0.24) | 0.2 |  |
| No record | 3.21(-5.83,12.26) | 0.47 |  |  | -0.08(-0.14,-0.03) | 0.005 |  |  | -1.18(-2.23,-0.13) | 0.03 |  |
| Hypertension |  |  | 0.36 |  |  |  | 0.01 |  |  |  | < 0.001 |
| No | 3.17(1.27,5.07) | 0.002 |  |  | -0.06(-0.09,-0.04) | <0.0001 |  |  | -0.35(-0.73,0.03) | 0.07 |  |
| Yes | 1.22(-2.02,4.46) | 0.45 |  |  | -0.12(-0.15,-0.08) | <0.0001 |  |  | -1.67(-2.43,-0.92) | <0.0001 |  |
| DM |  |  | 0.87 |  |  |  | 0.23 |  |  |  | 0.04 |
| No | 2.48(0.92,4.04) | 0.003 |  |  | -0.09(-0.11,-0.06) | <0.0001 |  |  | -0.72(-1.13,-0.31) | 0.001 |  |
| Yes | 2.38(-1.34,6.09) | 0.2 |  |  | -0.05(-0.11,0.00) | 0.03 |  |  | -2.2(-3.72,-0.67) | 0.01 |  |
| No recode | 5.02(-1.89,11.93) | 0.15 |  |  | -0.04(-0.08,0.00) | 0.03 |  |  | 0.5(-0.86,1.86) | 0.45 |  |
| Hyperlipidemia |  |  | 0.76 |  |  |  | 0.4 |  |  |  | 0.05 |
| No | 2.74(0.05,5.43) | 0.05 |  |  | -0.08(-0.11,-0.05) | <0.0001 |  |  | -0.55(-1.21,0.10) | 0.1 |  |
| Yes | 2.15(0.11,4.18) | 0.04 |  |  | -0.1(-0.12,-0.07) | <0.0001 |  |  | -1.16(-1.61,-0.71) | <0.0001 |  |
| CVD |  |  | 0.31 |  |  |  | 0.82 |  |  |  | 0.2 |
| No | 2.14(0.76,3.53) | 0.004 |  |  | -0.08(-0.11,-0.06) | <0.0001 |  |  | -0.73(-1.12,-0.33) | <0.001 |  |
| Yes | 4.97(-0.44,10.38) | 0.07 |  |  | -0.09(-0.17,-0.01) | 0.02 |  |  | -1.94(-3.97,0.09) | 0.06 |  |
| Cancer |  |  | 0.03 |  |  |  | 0.27 |  |  |  | 0.94 |
| No | 1.73(0.39,3.08) | 0.01 |  |  | -0.09(-0.12,-0.07) | <0.0001 |  |  | -1.06(-1.53,-0.60) | <0.0001 |  |
| Yes | 7.05(2.31,11.79) | 0.005 |  |  | -0.11(-0.14,-0.08) | <0.0001 |  |  | -1.02(-2.12,0.08) | 0.07 |  |

## Supplementary Figures


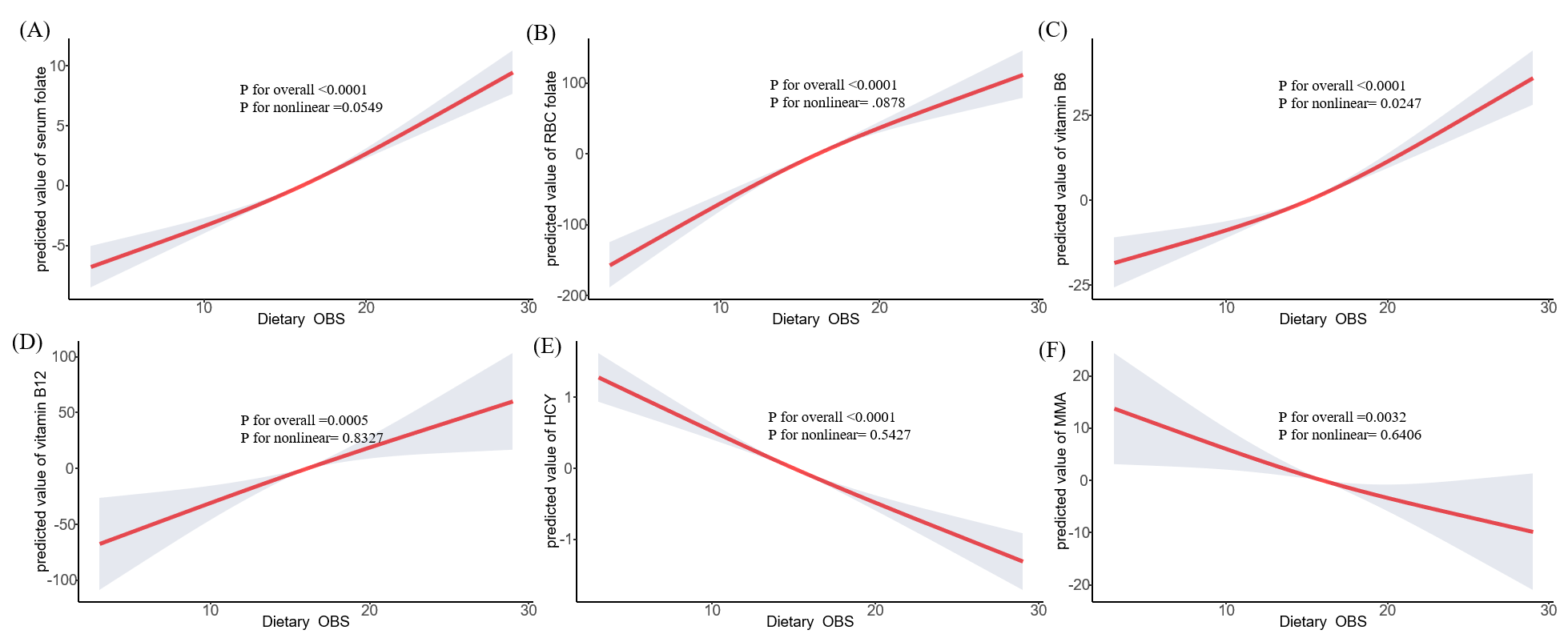
Figure S1: Restricted cubic spline analysis between dietary OBS and methylation cycle biomarkers.

RCS analysis adjusted for age, race, education, marital status, PIR, HEI, total energy intake, hypertension, DM, CVD, hyperlipidemia, and cancer.


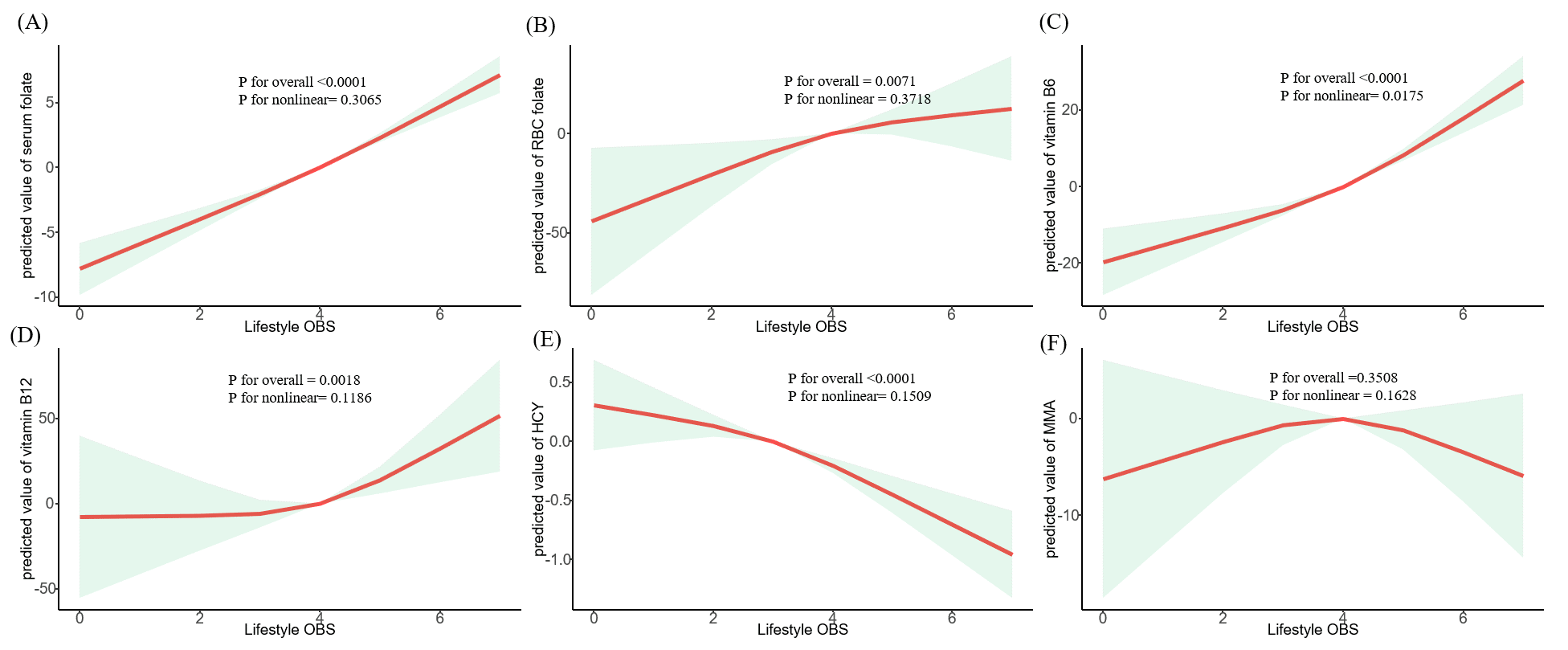
Figure S2: Restricted cubic spline analysis between lifestyle OBS and methylation cycle biomarkers.

RCS analysis adjusted for age, race, education, marital status, PIR, HEI, total energy intake, hypertension, DM, CVD, hyperlipidemia, and cancer.
